# *ApaI* polymorphism of vitamin D receptor affects health-related quality of life in patients with primary sclerosing cholangitis

**DOI:** 10.1371/journal.pone.0176264

**Published:** 2017-04-20

**Authors:** Agnieszka Kempinska-Podhorodecka, Malgorzata Milkiewicz, Dariusz Jabłonski, Piotr Milkiewicz, Ewa Wunsch

**Affiliations:** 1 Department of Medical Biology, Pomeranian Medical University in Szczecin, Szczecin, Poland; 2 Department of General and Endocrine Surgery, Medical University of Warsaw, Warsaw, Poland; 3 Liver and Internal Medicine Unit, Department of General, Transplant and Liver Surgery of the Medical University of Warsaw, Warsaw, Poland; 4 Translational Medicine Group, Pomeranian Medical University in Szczecin, Szczecin, Poland; Laval University, CANADA

## Abstract

**Background:**

Polymorphisms of vitamin D receptor (*VDR*) contribute to the pathogenesis of multiple autoimmune conditions.

**Methods:**

We investigated the incidence of *VDR* polymorphisms (*rs1544410-BsmI; rs7975232-ApaI; rs731236-TaqI)* in a group of patients with primary sclerosing cholangitis (PSC, n = 275) and in healthy controls (n = 376). Additionally, correlations of the *VDR* polymorphisms with clinical and biochemical factors of the disease were analysed.

**Results:**

The genotype and allele distributions of these polymorphisms in PSC patients were similar to those observed in controls. However, the *ApaI* polymorphism was associated with an impaired health-related quality of life (HRQoL). The generic SF-36 questionnaire showed that the *Role-Physical* (p = 0.01), *Role-Emotional* (p = 0.01), *Physical Component Summary* (p = 0.01) and *Mental Component Summary* (p = 0.003) scores were significantly affected. Similarly, the disease-specific questionnaires, PBC-40 and PBC-27, demonstrated that carriers of the C allele suffered from more severe *Itch* (p = 0.03 assessed by PBC-40 and PBC-27), more *Fatigue* (p = 0.02 assessed by PBC-40 and PBC-27) and *Impaired Cognitive Capacity* (p = 0.04 and p = 0.03). Correspondingly, individuals who were AA homozygotes (non-carriers of the C allele of ApaI) had higher summary scores for the *Physical* (p = 0.01) and *Mental Components* (p = 0.006) measured with SF-36. Moreover, they experienced less *itch* (p = 0.03) and less *Fatigue* (p = 0.03) and had better *Cognitive Abilities* (p = 0.04) as assessed by the PBC-40 and PBC-27 questionnaires. No associations between other *VDR* polymorphisms and clinical or laboratory findings were made.

**Conclusion:**

In summary, this study is the first to show that the *ApaI* polymorphisms in *VDR* may exert an effect on disease-related symptoms and quality of life in patients with PSC.

## Introduction

Primary sclerosing cholangitis (PSC), which frequently co-exists with inflammatory bowel disease, is a chronic cholestatic liver condition that affects both the small and large bile ducts. It occurs predominantly in males and often remains asymptomatic in the early stages of the disease. Nevertheless, progressing biliary tree damage ultimately leads to chronic cholestasis, recurrent cholangitis and liver cirrhosis in a high proportion of affected individuals [1], impairing the health-related quality of life (HRQoL)[2–4]. Moreover, patients with PSC are at an increased risk of cholangiocarcinoma, a primary biliary cancer with a fatal prognosis [5]. The effectiveness of pharmacological treatment remains controversial and insufficient [1,6], and liver transplantation remains the only curative option. Further recognition of its pathologic mechanisms may help to identify potential effective therapeutic targets. Unfortunately, the pathogenesis of PSC remains incompletely understood and is most likely related to the multimodal influences of inflammatory, autoimmune, genetic and infective factors[7]. Presumably, in genetically susceptible subjects, environmental factors trigger a pathological immune response that ultimately leads to lymphocyte migration, inflammation, and fibrotic damage of the biliary tree. While aiming to further understand PSC pathogenesis, several studies have focused on immunopathogenetic mechanisms, but the links between immunity and PSC remain unsatisfactorily explained.

1,25-Dihydroxyvitamin D_3_ (1,25(OH)_2_D_3_) exerts multiple immunomodulatory actions, and beyond its crucial role in mineral homeostasis, it is now believed to represent an important component of the immune response[8]. Strong evidence has shown that a disturbance in 1,25(OH)_2_D_3_ metabolism plays a role in the pathogenesis of several autoimmune diseases [9–15], including autoimmune liver disorders [16,17]. The effects of 1,25(OH)_2_D_3_ on target genes are mediated by a ligand-activated nuclear receptor, the vitamin D receptor (*VDR*) [18]. Several polymorphisms in the *VDR* gene have been described, but their effects on *VDR* function are poorly understood. Three of them—rs1544410 (*BsmI*), rs7975232 (*ApaI*), and rs731236 (*TaqI*)—have been linked to other chronic cholestatic conditions, including primary biliary cholangitis (PBC) [16,17,19–23], and our recent study has highlighted the association between the *BsmI* and *TaqI* variants and the disease severity [24].

In the study, we investigated the prevalence of *VDR* polymorphisms in a homogenous cohort of well-characterized Polish patients with PSC. Additionally, associations between *VDR* receptor polymorphisms were analysed in the context of health-related quality of life along with clinical as well as laboratory features of the disease.

## Materials and methods

### Patients

Two hundred and seventy-five patients (182 males, 93 females; median age at diagnosis 55 years, range 28–90 years) with PSC were recruited in two medical centres (Pomeranian Medical University, Szczecin, Poland and Medical University of Warsaw, Warsaw, Poland) between 2006 and 2015. The diagnosis of PSC was based on the MRCP/ERCP findings, per the EASL recommendations[25]. IgG4 cholangitis was excluded based on the laboratory and clinical profile. The demographic characteristic and main laboratory data of included subjects are presented in Table 1.

**Table 1:** Demographic data of analysed subjects.

Feature	PSC (n = 275)	Control group (n = 376)
**Age (median; range)**	55 (28–90)	27.8 (18–66)
**Gender (M/F)**	182/93	344/32
**Haemoglobin (median; range) IU/l**	13.2 (6.6–53.9)	N/A
**AST(median; range) IU/l**	92 (17–1628)	N/A
**ALT (median; range) IU/l**	130 (16–1411)	N/A
**ALP (median; range), IU/l**	354 (33–2061)	N/A
**GGT (median; range), IU/l**	332.5 (24–3102)	N/A
**Bilirubin (median; range), mg/dl**	1.3 (0.2–27)	N/A
**Cholesterol (median; range), mg/dl**	209 (72–871)	N/A
**Triglycerides (median; range), mg/dl**	79 (29–489)	N/A

AST: Aspartate aminotransferase; ALT: Alanine aminotransferase; ALP: Alkaline Phosphatase; GGT: Gamma-glutamyl transferase; *N/A—Not Applicable*

A cohort of 376 (age range 18–66 years) blood donors from the Regional Blood Donor Centre in Szczecin (Poland) was investigated. All subjects had a medical check-up, and a good state of health was a prerequisite to qualify for blood donation. Each participant provided his/her written informed consent. All consent records are deposited either in the Liver and Internal Medicine Unit, MUW, or in the Department of Medical Biology, PMU. The study protocol and consent procedure conform to the ethical guidelines of the 1975 Declaration of Helsinki (6th revision, 2008) and were approved by the Ethics Committee of Pomeranian Medical University.

### *VDR* genotyping

DNA from peripheral blood mononuclear cells was isolated using the DNeasy Blood & Tissue Kit (Qiagen). Oligonucleotide primers and TaqMan probes for *VDR* polymorphisms (rs7975232, rs15444410, rs731236) were designed and synthesized by Applied Biosystems (Assay ID: C_ 28977635_ 10, C_8716062_10, C_2404008_10, resp.). The fluorescence data were analysed with allelic discrimination 7500 Software v.2.0.2.

In addition to a nucleotide code, the description of the VDR genotype in the tables includes letters enclosed in square brackets that represent previously described nomenclature derived from a restriction-fragment length polymorphism (RFLP) analysis. The presence and absence of a restriction site are denoted with a lowercase and uppercase letter, respectively ([*b*, *B*] for *BsmI*; [*a*, *A*] for *ApaI*; [*t*, *T*] for *TaqI*) that also refers to a specific base change.

### HRQoL assessment

HRQoL is a multidimensional parameter that comprehensively assesses various aspects of human well-being, such as physical and cognitive capabilities, emotional status, and psychosocial adjustment, in the context of health and disease. HRQoL can be measured by generic or disease-specific questionnaires. In our study, we used one generic (Medical Outcome Study Short Form-36, SF-36) and two disease-specific (PBC-40 and PBC-27) tools. The SF-36 was designed in 1992 to measure the HRQoL in various populations and a wide variety of medical conditions to allow the possibility of comparing several health states[26]. The SF-36 includes 36 items divided into eight domains of physical health (*Physical Functioning*, *Role Limitation-Physical*, *Bodily Pain* and *General Health*) and mental health (*Vitality*, *Social Functioning*, *Role Limitation-Emotional* and *Mental Health*). Two summary scores, *Physical Component* and *Mental Component*, can also be calculated. Scale scores range between 0 (denoting the most impaired HQoL) and 100 (ideal well-being). License No. QM011392-QualityMetric CT133208/OP018661 was obtained for the use of the SF-36 questionnaire in this study.

The PBC-40 questionnaire and its simplified form, PBC-27, were designed for the assessment of disease-specific symptoms among patients with PBC [27,28]. Both questionnaires were recently validated by our group for use in PSC [2]. PBC-40 consists 40 questions in 5 domains including *Cognition*, *Itch*, *Fatigue*, *Social-Emotional* and *Other Symptoms* that are assessed using a five-point scale (1 = never to 5 = always), with higher scores denoting a greater symptom impact and poorer HRQoL. The possible range of each domain is as follows: *Symptom* domain (7–35), Itch (3–15), *Fatigue* (11–55), *Cognitive* (6–30), *Social* and *Emotional* (13–65) points. In the PBC-27 questionnaire, 27 items are grouped into 7 domains as follows: *Other Symptoms* (possible range: 3–15 points), *Dryness* (2–10 points), *Itch* (3–15 points), *Fatigue* (8–40 points), *Cognitive* (5–25 points), *Emotional* (3–15 points) and *Social* (3–15 points), with the same 5-point scale of evaluation.

### Statistics

All statistical analyses were performed using the Stat-View-5 Software (SAS Institute, Cary, NC, US). The genotype and allele frequencies were compared between patients and controls using Fisher`s exact probability test. The odds ratio (OR) and 95% confidence interval (CI) for each variable were also estimated. The analysis of genotype frequency in regards to the clinical characteristics and HRQoL assessment of PSC patients was performed using ANOVA with Fisher's protected least significant difference (Fisher's. PLSD). Data were evaluated as the mean ± standard deviation (SD) for continuous variables. A two-sided significance level of 0.05 was considered to indicate a statistically significant difference.

## Results

No significant differences in the genotype or allelic frequencies of the *VDR* polymorphisms between the PBC patients and healthy controls were seen (Tables 2 and 3). However, one of the examined *VDR* polymorphisms, the *Apal* variant, showed a negative effect on the patients’ well-being as measured with the generic and disease-specific questionnaires.

**Table 2:** Genotype counts for *VDR* polymorphisms (rs1544410, rs7975232, rs731236) in PSC patients and in controls.

Genotype	PSC (%) (n = 275)	Controls (%) (n = 376)	P* Value	*X*^*2*^	*OR (95% CI)*
**rs1544410 (*BsmI*)**					
**AA [*BB*]**	40 (14.5%)	44 (11.7%)	*0*.*3*	1.14	1.28 (0.8–2.0)
**GA [*bB*]**	121 (44.0%)	160 (42.6.2%)	*0*.*7*	0.1	1.06 (0.8–1.5)
**GG [*bb*]**	114 (41.5%)	172 (45.7%)	*0*.*3*	1.2	0.8 (0.6–1.2)
**rs7975232 (*ApaI*)**					
**AA [*AA*]**	67 (24.4%)	74 (19.7%)	*0*.*2*	2.1	1.3 (0.9–1.9)
**AC [*aA*]**	124 (45.1%)	196 (52.1%)	*0*.*8*	3.1	0.7 (0.6–1.03)
**CC [*aa*]**	84 (30.5%)	106 (28.2%)	*0*.*5*	0.4	1.1 (0.8–1.6)
**rs731236 (*TaqI*)**					
**TT [*TT*]**	116 (42.2%)	172 (45.7%)	*0*.*5*	0.6	0.9 (0.6–1.2)
**TC [*Tt*]**	124 (45.1%)	160 (42.6%)	*0*.*5*	0.4	1.1 (0.8–1.5)
**CC [*tt*]**	35 (12.7%)	44 (11.7%)	*0*.*7*	0.2	1.1 (0.7–1.8)

* Fisher`s exact probability test; Chi-squared test for categorical variables

PSC: Primary Sclerosing Cholangitis; OR: odds ratio; CI: confidence interval.

**Table 3:** Allele association for *VDR* in patients with PSC and control subjects.

SNP	Allele	PSC (n = 275)	Controls (n = 376)	P* Value	*X*^*2*^	OR (95%CI)
**rs1544410** **(*BsmI)***	A/G [*B/b*]	201 (36.5%)/349 (63.5%)	248 (33%)/504 (67%)	*0*.*2*	1.8	1.2 (0.9–1.4)
**rs7975232** ***(ApaI)***	A/C [*A/a*]	258 (46.9%)/292 (53.1%)	344 (45.7%)/408 (44.3%)	*0*.*6*	6.9	1.1 (0.8–1.3)
**rs731236** ***(TaqI)***	C/T [t/T]	194 (35.3%)/356 (64.7%)	248 (33%)/504 (67%)	*0*.*4*	0.7	1.1 (0.9–1.4)

* Fisher`s exact probability test;

PSC: Primary Sclerosing Cholangitis; OR: odds ratio; CI: confidence interval.

Data for HRQoL are available for 167 patients. Significant negative associations between the C [a] allele of rs7975232 and 4 domains of SF-36 were found. These included the *Role-Physical* (*P* = 0.01), *Role-Emotional* (*P* = 0.01), *Physical Component Summary* (*P* = 0.01), and *Mental Component Summary* (*P* = 0.003) scores. Similarly, the disease-specific questionnaires, including PBC-40 and PBC-27, demonstrated that subjects who were C [a] carriers of rs7975232 suffered from more severe *Itch* (*P* = 0.03 and *P* = 0.03) and *Fatigue* (*P* = 0.02 and *P* = 0.02) and an *Impaired Cognitive Capacity* (*P* = 0.04 and *P* = 0.03), respectively. These data are summarized in Table 4.

**Table 4:** Allelic analysis of rs1544410, rs7975232 and rs731236 in relation to SF-36, PBC-40 and PBC-27 domains.

**SF-36**	**rs1544410 (*BsmI*)**			**rs7975232 (*ApaI*)**			**rs731236 (*TaqI*)**		
	***A [B]***	***G [b]***	P*	***A [A]***	***C [a]***	P*	***[T]***	***[t]***	P*
***Physical functioning***	85.2±4.2	81.2±1.9	*NS*	87.7±3.1	80.7±2.1	*NS*	79.0±2.9	85.2±2,2	*NS*
***Role-Physical***	73.0±7.2	61.8±3.5	*NS*	77.0±5.7	59.4±3.7	***0*.*01***	57.2±5.0	68.8±4.0	*NS*
***Bodily Pain***	73.4±5.6	72.9±2.5	*NS*	80.2±4.2	70.7±2.6	*NS*	70.5±3.7	74.9±2.9	*NS*
***General Health***	50.3±5.8	48.0±1.8	*NS*	53.5±4.0	46.7±1.9	*NS*	46.8±2.5	49.6±2.5	*NS*
***Vitality***	54.7±4.6	54.6±1.7	*NS*	58.7±3.5	53.4±1.7	*NS*	53.1±2.4	55.9±2.1	*NS*
***Social Functioning***	68.7±5.2	64.6±2.0	*NS*	72.1±3.9	63.1±2.2	*NS*	61.8±2.9	68.0±2.6	*NS*
***Role-Emotional***	83.5±6.9	72.3±3.2	*NS*	87.1±5.1	69.8±3.1	***0*.*01***	67.8±4.4	78.9±3.8	*NS*
***Mental Health***	68.6±3.8	64.9±1.7	*NS*	69.3±3.1	64.3±1.7	*NS*	62.2±2.4	68.2±1.9	*NS*
***Physical Component Summary***	67.9±4.6	64.9±1.8	*NS*	73.2±3.5	62.9±1.9	***0*.*01***	62.6±2.6	67.5±2.3	*NS*
***Mental Component Summary***	65.9±4.3	61.5±1.8	*NS*	71.3±3.3	59.3±1.9	***0*.*003***	58.6±2.5	65.0±2.2	*NS*
**PBC-40**	**rs1544410 (*BsmI*)**			**rs7975232 (*ApaI*)**			**rs731236 (*TaqI*)**		
	***A [B]***	***G [b]***	P*	***A [A]***	***C [a]***	P*	***[T]***	***[t]***	P*
***Other Symptom***	13.7±0.8	12.6±0.4	*NS*	12.5±0.7	12.9±0.4	*NS*	12.8±0.5	12.7±0.5	*NS*
***Itch***	3.8±0.6	4.7±0.3	*NS*	3.5±0.5	5.0±0.4	***0*.*03***	5.1±0.5	4.3±0.4	*NS*
***Fatigue***	22.0±1.6	24.5±0.8	*NS*	21.1±1.3	25.1±0.9	***0*.*02***	24.8±1.1	23.5±1.0	*NS*
***Cognitive***	10.1±0.8	10.9±0.4	*NS*	9.3±0.6	11.2±0.4	***0*.*04***	10.9±0.5	10.5±0.5	*NS*
***Social and Emotional***	27.0±2.1	29.8±0.8	*NS*	26.4±1.5	30.3±0.9	***0*.*03***	30.7±1.1	28.2±1.0	*NS*
**PBC-27**	**rs1544410 (*BsmI*)**			**rs7975232 (*ApaI*)**			**rs731236 (*TaqI*)**		
	***A [B]***	***G [b]***	P*	***A [A]***	***C [a]***	P*	***[T]***	***[t]***	P*
***Other Symptom***	6.4±0.5	6.2±0.2	*NS*	6.0±0.4	6.3±0.2	*NS*	6.4±0.3	6.1±0.2	*NS*
***Dryness***	3.9±0.3	3.9±0.1	*NS*	3.6±0.2	4.1±0.1	*NS*	4.1±0.2	3.9±0.1	*NS*
***Itch***	3.8±0.6	4.7±0.3	*NS*	3.4±0.5	5.0±0.3	***0*.*03***	5.0±0.5	4.2±0.3	*NS*
***Fatigue***	16.6±1.1	18.6±0.6	*NS*	16.0±0.9	19.0±0.6	***0*.*02***	19.1±0.8	17.6±0.7	*NS*
***Cognitive***	8.3±0.6	9.1±0.4	*NS*	7.7±0.5	9.4±0.4	***0*.*03***	9.2±0.5	8.8±0.4	*NS*
***Emotional***	6.4±0.5	6.7±0.2	*NS*	6.0±0.4	6.8±0.3	*NS*	6.8±0.3	6.6±0.3	*NS*
***Social***	6.2±0.7	6.9±0.2	*NS*	5.9±0.5	7.1±0.3	*NS*	7.2±0.3	6.5±0.4	*NS*

* ANOVA with Fisher's protected least significant difference (PLSD); NS: not significant.

Correspondingly, the AA homozygotes of the rs7975232 who did not have the C [a] allele had significantly higher *Physical* (*P* = 0.01 vs CC, and *P* = 0.04 vs AC) and *Mental Component Summary* scores as measured with SF-36 (*P* = 0.006 vs CC, and *P* = 0.009 vs AC), respectively (Table 5). No correlations were found between the genotypes and allelic analyses of rs1544410 (*BsmI*) or rs731236 (*TaqI*) and the quality of life features using the SF-36, PBC-40 and PBC-27 questionnaires (Table 4).

**Table 5:** Relationship between the rs7975232 VDR polymorphisms and features of the SF-36, PBC-40 and PBC-27 questionnaires.

SF-36	rs7975232 (*ApaI*)					
	AA [*AA*]	AC [*Aa*]	CC [*aa*]	P* AA vs AC	P* AA vs CC	P* AC vs CC
***Physical Functioning***	87.7±3.6	81.1±2.7	80.2±3.5	*NS*	*NS*	*NS*
***Role-Physical***	77.0±5.8	56.0±4.9	64.3±5.7	***0*.*01***	*NS*	*NS*
***Bodily Pain***	80.2±4.2	69.3±3.5	72.7±4.1	*0*.*06*	*NS*	*NS*
***General Health***	53.6±4.0	47.6±2.5	45.4±3.3	*NS*	*NS*	*NS*
***Vitality***	58.7±3.5	52.9±2.1	54.0±3.0	*NS*	*NS*	*NS*
***Social Functioning***	72.1±3.9	62.7±3.0	63.6±3.4	*0*.*06*	*NS*	*NS*
***Role-Emotional***	87.1±5.1	71.3±4.5	67.7±5.0	***0*.*03***	***0*.*01***	*NS*
***Mental Health***	69.3±3.1	66.1±2.2	61.7±3.0	*NS*	*0*.*07*	*NS*
***Physical Component Summary***	73.2±3.5	62.2±2.5	63.9±3.1	***0*.*01***	***0*.*04***	*NS*
***Mental Component Summary***	71.3±3.3	59.5±2.4	59.3±3.1	***0*.*006***	***0*.*009***	*NS*
**PBC-40**						
***Other Symptom***	12.5±0.7	12.8±0.5	13.0±0.7	*NS*	*NS*	*NS*
***Itch***	3.4±0.5	5.1±0.5	4.8±0.6	***0*.*03***	*NS*	*NS*
***Fatigue***	21.1±1.2	25.3±1.1	24.8±1.5	***0*.*03***	*0*.*07*	*NS*
***Cognitive***	9.3±0.6	11.2±06	11.1±07	***0*.*04***	*0*.*07*	*NS*
***Social and Emotional***	26.3±1.5	30.4±1.2	30.2±1.4	***0*.*04***	*0*.*08*	*NS*
**PBC-27**						
***Other Symptom***	6.0±04	6.3±0.3	6.3±0.4	*NS*	*NS*	*NS*
***Dryness***	3.6±0.2	4.0±0.2	4.2±0.3	*NS*	*NS*	*NS*
***Itch***	3.5±0.5	5.1±0.5	4.8±0.6	***0*.*03***	*NS*	*NS*
***Fatigue***	16.0±0.9	18.9±0.8	19.1±1.1	***0*.*04***	***0*.*03***	*NS*
***Cognitive***	7.7±0.5	9.4±0.5	9.3±0.6	***0*.*04***	*0*.*07*	*NS*
***Emotional***	6.0±0.4	7.0±0.3	6.7±0.4	*NS*	*NS*	*NS*
***Social***	6.0±0.5	7.3±0.4	6.9±0.4	***0*.*05***	*NS*	*NS*

*Fisher's. PLSD; NS: not significant.

The presence of these polymorphisms did not correlate with analysed clinical features such as gender, age and cirrhosis at presentation or liver biochemistry at diagnosis (S1 Table).

## Discussion

In this study, we have analysed the prevalence of three common *VDR* polymorphisms (*Apal*-rs7975232, *Bsml*-rs15444410, *Taql*-rs731236) and investigated their potential relationships with the severity of disease-related symptoms in a well characterized cohort of Polish patients with PSC. Despite similar distributions of the *VDR* variants in patients with PSC and in healthy subjects, our study clearly indicated that the *VDR* polymorphisms impact the clinical phenotype of PSC patients. We have shown that the *Apal* variant of the *VDR* gene profoundly impairs well-being among patients with PSC as measured with the general and disease-specific questionnaires. *Apal* allele *a* was associated with a worse HRQoL as measured by generic SF-36 in the following domains: *Role Limitation-Physical*, *Role Limitation-Emotional* and the *Physical* and *Mental Component Summaries*. Moreover, the analysis of the PBC-40/PBC-27 domains showed that HRQoL scores for the carriers of *Apal* allele *a* were almost all impaired; the impaired scores included *Itch*, *Fatigue* and *Cognitive* in both questionnaires and the *Social and Emotional* domain in PBC-40. We obtained similar results when analysing the genotype profiles; the heterozygotes and homozygotes carrying *Apal* allele *a* showed impaired well-being scores in the aforementioned domains of SF-36 and PBC-40/PBC-27.

Impaired quality of life is often associated with symptoms such as chronic fatigue and is quite frequently seen in patients with chronic cholestasis [29]. Few reports have already indicated the negative impact of PSC on HRQoL [3,4]. In our previous study, we observed an impairment in quality of life for patients with PSC compared to healthy individuals, and our data highlighted a significant impact of female gender in predicting worse quality of life [2]. Our current project increases our knowledge on the impact of genetic variations in PSC on patients’ well-being, as this is the first study the focus on HRQoL assessment in this context. Our results suggest that although the analysed *VDR* variants do not increase the susceptibility to PSC, they may have an impact on the severity of disease-related symptoms. The mechanistic background of this association remains difficult to explain because the functional effects of *VDR* polymorphisms are still poorly understood. Because the location of *Apal* polymorphism is intronic, it might affect alternative splicing of the *VDR* mRNA or be relevant as an enhancer that augments the transcription of an associated gene. It is also possible that *Apal* may be a genetic marker for other truly functional variations elsewhere in the *VDR* gene that are in linkage disequilibrium with the identified polymorphism[30].

Interestingly, the *ApaI* polymorphism was associated with impaired cognitive function in elderly Chinese subjects [31] and with cognitive impairment and depression in elderly Dutch patients [32]. Another functional polymorphism of *VDR*, namely, *Fokl*, was found to be associated with cognitive decline in American patients with Parkinson’s disease [33]. Moreover, correction of vitamin D deficiency exerted an ameliorating effect on chronic fatigue in a large cohort of more than 170 subjects presenting with this symptom to their general practitioners [34], and Vitamin D replacement significantly improved depressive symptoms in women with chronic liver diseases [35].

Although our study does not fully elucidate the mechanism that underlies the observed association, our data may be of clinical relevance. In the liver, *VDR* is expressed in non-parenchymal cells and biliary epithelial cells[36]. After binding its ligand, VDR forms a heterodimer with the retinoid X receptor (RXR) to modulate divergent pathways ranging from calcium metabolism to immune system homoeostasis. Furthermore, 1,25(OH)_2_D_3_, lithocholic acid and its metabolites have been shown to act as *VDR* ligands [37]. Moreover, VDR-related pathways are engaged in the regulation of bile acid synthesis and detoxification[38,39]. These findings, and especially the data that show how common 1,25(OH)_2_D_3_ deficiency is in autoimmune conditions, suggest that dysfunction of *VDR* may play a potential role in cholestatic liver injury. Thus, several studies have been performed regarding homeostasis of vitamin D in chronic cholestasis, but the vast majority have focused on PBC, while little has been done in relation to PSC. PBC genetic studies of the *VDR* have repeatedly indicated the association of the *BsmI* polymorphism with susceptibility to PBC[17,20–22,40]. Moreover, our recent study indicated the relationship between the *BsmI* and *TaqI* polymorphisms of the *VDR* gene and the presence of liver cirrhosis and advanced fibrosis[24].

The data regarding vitamin D-VDR signalling in PSC are more scarce. Most available analyses concerning PSC specifically focus on the serum levels of 1,25(OH)_2_D_3_. To date, there are no available studies in the setting of the *VDR* gene variability in PSC. Our study is the first analysis of three polymorphisms that have been previously indicated as risk factors of PBC and other autoimmune conditions. We showed that there is no relationship between *VDR* variants and susceptibility to PSC. These findings are in accordance with previous genome-wide association studies (GWAS), which recognize the strongest genetic risk for PSC within the major histocompatibility complex (MHC) and within several other loci that contain genes that regulate immune self-recognition and adaptive immunity, but not within the *VDR* gene[41].

Two decades ago, Jorgensen R.A. *et al*. found vitamin D deficiency among patients with PSC, particularly in patients with advanced disease who were evaluated for liver transplantation. In the pretransplantation group, lower levels of 1,25(OH)_2_D_3_ were observed in over half of patients, compared to 14% of subjects in the less advanced clinical condition[42]. Further studies have shown that vitamin D deficiency is commonly seen in patients with chronic liver disease regardless of the underlying aetiology of the liver injury and that it correlates with fibrosis progression[43–45]. Moreover, vitamin D deficiency has been proven to impair the course of liver disease and prognosis [46–48]. The evidence deriving from *in vitro* and animal studies suggest that supplementation of vitamin D may exert beneficial effects in PSC. A study by Hochrath *et al*. demonstrated that vitamin D diminishes hepatic inflammation in Abcb4-/- mice, a reproducible animal model of sclerosing cholangitis[49]. Moreover, vitamin D inhibits activation and proliferation of murine hepatic stellate cells, which produce the extracellular matrix proteins that are deposited in liver fibrosis. These studies suggest that 1,25(OH)_2_D_3_ is potentially an attractive therapeutic agent that may ameliorate cholestatic liver injury. In view of these and our findings, further studies should focus on the potential influence of vitamin D on laboratory parameters as well as disease-related symptoms.

The fact that we did not measure the serum concentration of 1,25-dihydroxyvitamin D can be considered a limitation of our data. However, our recent study clearly demonstrated a significant reduction in Vitamin D receptor mRNA and protein expression in liver tissues from patients with PSC [50]. This phenomenon may clearly decrease the hepatic availability of Vitamin D followed by a limitation to its cellular effects.

## Conclusions

In conclusion, our study is the first to address the relationship between polymorphisms within the *VDR* gene and the clinical characteristics of PSC. We observed a profound effect by the *Apal* variants on disease-related symptoms in the studied cohort. The explanation of these findings is hindered by the unknown functional effects of *VDR* gene variations. Further studies are needed to investigate the pathophysiological background of the observed association and to check if the modulation of vitamin D-VDR signalling exerts beneficial effects on the clinical course of the disease.

## Supporting information

S1 Table Clinical and laboratory date depending an analyzed polymorphisms. (DOC)

## References

[1] Hirschfield GM, Karlsen TH, Lindor KD, Adams DH (2013) Primary sclerosing cholangitis. Lancet 382: 1587–1599. 10.1016/S0140-6736(13)60096-3 23810223

[2] Raszeja-Wyszomirska J, Wunsch E, Krawczyk M, Rigopoulou EI, Bogdanos D, Milkiewicz P (2015) Prospective evaluation of PBC-specific health-related quality of life questionnaires in patients with primary sclerosing cholangitis. Liver Int 35: 1764–1771. 10.1111/liv.12730 25388280

[3] Cheung AC, Patel H, Meza-Cardona J, Cino M, Sockalingam S, Hirschfield GM (2016) Factors that Influence Health-Related Quality of Life in Patients with Primary Sclerosing Cholangitis. Dig Dis Sci 61: 1692–1699. 10.1007/s10620-015-4013-1 26743764

[4] Benito d, V, Rahman M, Lindkvist B, Bjornsson E, Chapman R, Kalaitzakis E (2012) Factors that reduce health-related quality of life in patients with primary sclerosing cholangitis. Clin Gastroenterol Hepatol 10: 769–775. 10.1016/j.cgh.2012.01.025 22343690

[5] Milkiewicz P, Wunsch E (2011) Primary sclerosing cholangitis. Recent Results Cancer Res 185: 117–133. 10.1007/978-3-642-03503-6_7 21822823

[6] Wunsch E, Trottier J, Milkiewicz M, Raszeja-Wyszomirska J, Hirschfield GM, Barbier O, et al (2014) Prospective evaluation of ursodeoxycholic acid withdrawal in patients with primary sclerosing cholangitis. Hepatology 60: 931–940. 10.1002/hep.27074 24519384

[7] Eaton JE, Talwalkar JA, Lazaridis KN, Gores GJ, Lindor KD (2013) Pathogenesis of primary sclerosing cholangitis and advances in diagnosis and management. Gastroenterology 145: 521–536. 10.1053/j.gastro.2013.06.052 23827861 PMC3815445

[8] Peelen E, Knippenberg S, Muris AH, Thewissen M, Smolders J, Tervaert JW, et al (2011) Effects of vitamin D on the peripheral adaptive immune system: a review. Autoimmun Rev 10: 733–743. 10.1016/j.autrev.2011.05.002 21621002

[9] Rosen Y, Daich J, Soliman I, Brathwaite E, Shoenfeld Y (2016) Vitamin D and autoimmunity. Scand J Rheumatol 45: 439–447. 10.3109/03009742.2016.1151072 27191042

[10] Agmon-Levin N, Blank M, Zandman-Goddard G, Orbach H, Meroni PL, Tincani A, et al (2011) Vitamin D: an instrumental factor in the anti-phospholipid syndrome by inhibition of tissue factor expression. Ann Rheum Dis 70: 145–150. 10.1136/ard.2010.134817 20980705

[11] Amital H, Szekanecz Z, Szucs G, Danko K, Nagy E, Csepany T, et al (2010) Serum concentrations of 25-OH vitamin D in patients with systemic lupus erythematosus (SLE) are inversely related to disease activity: is it time to routinely supplement patients with SLE with vitamin D? Ann Rheum Dis 69: 1155–1157. 10.1136/ard.2009.120329 20439290

[12] Arnson Y, Amital H, Agmon-Levin N, Alon D, Sanchez-Castanon M, Lopez-Hoyos M, et al (2011) Serum 25-OH vitamin D concentrations are linked with various clinical aspects in patients with systemic sclerosis: a retrospective cohort study and review of the literature. Autoimmun Rev 10: 490–494. 10.1016/j.autrev.2011.02.002 21320645

[13] Ishikawa LL, Colavite PM, Fraga-Silva TF, Mimura LA, Franca TG, Zorzella-Pezavento SF, et al (2016) Vitamin D Deficiency and Rheumatoid Arthritis. Clin Rev Allergy Immunol. 10.1007/s12016-016-8577-0 27484684

[14] Kivity S, Agmon-Levin N, Zisappl M, Shapira Y, Nagy EV, Danko K, et al (2011) Vitamin D and autoimmune thyroid diseases. Cell Mol Immunol 8: 243–247. 10.1038/cmi.2010.73 21278761 PMC4012880

[15] Lerner A, Shapira Y, Agmon-Levin N, Pacht A, Ben-Ami SD, Lopez HM, et al (2012) The clinical significance of 25OH-Vitamin D status in celiac disease. Clin Rev Allergy Immunol 42: 322–330. 10.1007/s12016-010-8237-8 21210250

[16] Fan LY, Tu XQ, Zhu Y, Pfeiffer T, Feltens R, Stoecker W, et al (2005) Genetic association of cytokines polymorphisms with autoimmune hepatitis and primary biliary cirrhosis in the Chinese. World J Gastroenterol 11: 2768–2772. 10.3748/wjg.v11.i18.2768 15884119 PMC4305913

[17] Vogel A, Strassburg CP, Manns MP (2002) Genetic association of vitamin D receptor polymorphisms with primary biliary cirrhosis and autoimmune hepatitis. Hepatology 35: 126–131. 10.1053/jhep.2002.30084 11786968

[18] Carlberg C (2003) Current understanding of the function of the nuclear vitamin D receptor in response to its natural and synthetic ligands. Recent Results Cancer Res 164: 29–42. 12899512 10.1007/978-3-642-55580-0_2

[19] Guardiola J, Xiol X, Nolla JM (2000) Influence of vitamin D receptor gene polymorphism on bone mineral density in primary biliary cirrhosis. Gastroenterology 119: 599–600. S0016508500012555 [pii]. 10960276 10.1053/gast.2000.16155

[20] Halmos B, Szalay F, Cserniczky T, Nemesanszky E, Lakatos P, Barlage S, et al (2000) Association of primary biliary cirrhosis with vitamin D receptor BsmI genotype polymorphism in a Hungarian population. Dig Dis Sci 45: 1091–1095. 10877221 10.1023/a:1005581414918

[21] Lakatos LP, Bajnok E, Hegedus D, Toth T, Lakatos P, Szalay F (2002) Vitamin D receptor, oestrogen receptor-alpha gene and interleukin-1 receptor antagonist gene polymorphisms in Hungarian patients with primary biliary cirrhosis. Eur J Gastroenterol Hepatol 14: 733–740. 12169981 10.1097/00042737-200207000-00004

[22] Resnick RH, Chopra S (2000) Vitamin D receptor gene analysis in primary biliary cirrhosis. Gastroenterology 119: 1805. S0016508500504442 [pii]. 10.1016/s0016-5085(00)70039-4 11187436

[23] Springer JE, Cole DE, Rubin LA, Cauch-Dudek K, Harewood L, Evrovski J, et al (2000) Vitamin D-receptor genotypes as independent genetic predictors of decreased bone mineral density in primary biliary cirrhosis. Gastroenterology 118: 145–151. S0016508500563379 [pii]. 10611163 10.1016/s0016-5085(00)70423-9

[24] Kempinska-Podhorecka A, Wunsch E, Jarowicz T, Raszeja-Wyszomirska J, Loniewska B, Kaczmarczyk M, et al (2012) Vitamin d receptor polymorphisms predispose to primary biliary cirrhosis and severity of the disease in polish population. Gastroenterol Res Pract 2012: 408723 10.1155/2012/408723 22690210 PMC3368329

[25] European Association For The Study Of The Liver (2009) EASL Clinical Practice Guidelines: management of cholestatic liver diseases. Journal of hepatology 51: 237–267. 10.1016/j.jhep.2009.04.009 19501929

[26] Ware JE Jr., Sherbourne CD (1992) The MOS 36-item short-form health survey (SF-36). I. Conceptual framework and item selection. Med Care 30: 473–483. 1593914

[27] Montali L, Tanaka A, Riva P, Takahashi H, Cocchi C, Ueno Y, et al (2010) A short version of a HRQoL questionnaire for Italian and Japanese patients with Primary Biliary Cirrhosis. Dig Liver Dis 42: 718–723. 10.1016/j.dld.2010.01.004 20163995

[28] Newton JL, Bhala N, Burt J, Jones DE (2006) Characterisation of the associations and impact of symptoms in primary biliary cirrhosis using a disease specific quality of life measure. J Hepatol 44: 776–783. 10.1016/j.jhep.2005.12.012 16487619

[29] Milkiewicz P, Heathcote EJ (2004) Fatigue in chronic cholestasis. Gut 53: 475–477. 10.1136/gut.2003.025155 15016736 PMC1774021

[30] Uitterlinden AG, Fang Y, Van Meurs JB, Pols HA, Van Leeuwen JP (2004) Genetics and biology of vitamin D receptor polymorphisms. Gene 338: 143–156. 10.1016/j.gene.2004.05.014 15315818

[31] Keyimu K, Zhou XH, Miao HJ, Zou T (2014) Relationship between vitamin D receptor gene polymorphism and mild cognitive impairment in elderly Uygur people. Int J Clin Exp Med 7: 5282–5288. 25664032 PMC4307479

[32] Kuningas M, Mooijaart SP, Jolles J, Slagboom PE, Westendorp RG, van HD (2009) VDR gene variants associate with cognitive function and depressive symptoms in old age. Neurobiol Aging 30: 466–473. 10.1016/j.neurobiolaging.2007.07.001 17714831

[33] Gatto NM, Paul KC, Sinsheimer JS, Bronstein JM, Bordelon Y, Rausch R, et al (2016) Vitamin D receptor gene polymorphisms and cognitive decline in Parkinson's disease. J Neurol Sci 370: 100–106. 10.1016/j.jns.2016.09.013 27772736 PMC5325129

[34] Roy S, Sherman A, Monari-Sparks MJ, Schweiker O, Hunter K (2014) Correction of Low Vitamin D Improves Fatigue: Effect of Correction of Low Vitamin D in Fatigue Study (EViDiF Study). N Am J Med Sci 6: 396–402. 10.4103/1947-2714.139291 25210673 PMC4158648

[35] Stokes CS, Grunhage F, Baus C, Volmer DA, Wagenpfeil S, Riemenschneider M, et al (2016) Vitamin D supplementation reduces depressive symptoms in patients with chronic liver disease. Clin Nutr 35: 950–957. 10.1016/j.clnu.2015.07.004 26212170

[36] Gascon-Barre M, Demers C, Mirshahi A, Neron S, Zalzal S, Nanci A (2003) The normal liver harbors the vitamin D nuclear receptor in nonparenchymal and biliary epithelial cells. Hepatology 37: 1034–1042. 10.1053/jhep.2003.50176 12717384

[37] Makishima M, Lu TT, Xie W, Whitfield GK, Domoto H, Evans RM, et al (2002) Vitamin D receptor as an intestinal bile acid sensor. Science 296: 1313–1316. 10.1126/science.1070477 12016314

[38] Jiang W, Miyamoto T, Kakizawa T, Nishio SI, Oiwa A, Takeda T, et al (2006) Inhibition of LXRalpha signaling by vitamin D receptor: possible role of VDR in bile acid synthesis. Biochem Biophys Res Commun 351: 176–184. 10.1016/j.bbrc.2006.10.027 17054913

[39] Schmidt DR, Holmstrom SR, Fon TK, Bookout AL, Kliewer SA, Mangelsdorf DJ (2010) Regulation of bile acid synthesis by fat-soluble vitamins A and D. J Biol Chem 285: 14486–14494. 10.1074/jbc.M110.116004 20233723 PMC2863217

[40] Tanaka A, Nezu S, Uegaki S, Kikuchi K, Shibuya A, Miyakawa H, et al (2009) Vitamin D receptor polymorphisms are associated with increased susceptibility to primary biliary cirrhosis in Japanese and Italian populations. J Hepatol 50: 1202–1209. 10.1016/j.jhep.2009.01.015 19376604

[41] Karlsen TH, Franke A, Melum E, Kaser A, Hov JR, Balschun T, et al (2010) Genome-wide association analysis in primary sclerosing cholangitis. Gastroenterology 138: 1102–1111. 10.1053/j.gastro.2009.11.046 19944697

[42] Jorgensen RA, Lindor KD, Sartin JS, LaRusso NF, Wiesner RH (1995) Serum lipid and fat-soluble vitamin levels in primary sclerosing cholangitis. J Clin Gastroenterol 20: 215–219. 7797830 10.1097/00004836-199504000-00011

[43] Arteh J, Narra S, Nair S (2010) Prevalence of vitamin D deficiency in chronic liver disease. Dig Dis Sci 55: 2624–2628. 10.1007/s10620-009-1069-9 19960254

[44] Lim LY, Chalasani N (2012) Vitamin d deficiency in patients with chronic liver disease and cirrhosis. Curr Gastroenterol Rep 14: 67–73. 10.1007/s11894-011-0231-7 22113744

[45] Stokes CS, Volmer DA, Grunhage F, Lammert F (2013) Vitamin D in chronic liver disease. Liver Int 33: 338–352. 10.1111/liv.12106 23402606

[46] Petta S, Camma C, Scazzone C, Tripodo C, Di M, V, Bono A, et al (2010) Low vitamin D serum level is related to severe fibrosis and low responsiveness to interferon-based therapy in genotype 1 chronic hepatitis C. Hepatology 51: 1158–1167. 10.1002/hep.23489 20162613

[47] Putz-Bankuti C, Pilz S, Stojakovic T, Scharnagl H, Pieber TR, Trauner M, et al (2012) Association of 25-hydroxyvitamin D levels with liver dysfunction and mortality in chronic liver disease. Liver Int 32: 845–851. 10.1111/j.1478-3231.2011.02735.x 22222013

[48] Trepo E, Ouziel R, Pradat P, Momozawa Y, Quertinmont E, Gervy C, et al (2013) Marked 25-hydroxyvitamin D deficiency is associated with poor prognosis in patients with alcoholic liver disease. J Hepatol 59: 344–350. 10.1016/j.jhep.2013.03.024 23557869

[49] Hochrath K, Stokes CS, Geisel J, Pollheimer MJ, Fickert P, Dooley S, et al (2014) Vitamin D modulates biliary fibrosis in ABCB4-deficient mice. Hepatol Int 8: 443–452. 10.1007/s12072-014-9548-2 25191532 PMC4148166

[50] Kempinska-Podhorodecka A, Milkiewicz M, Wasik U, Ligocka J, Zawadzki M, Krawczyk M, et al (2017) Decreased Expression of Vitamin D Receptor Affects an Immune Response in Primary Biliary Cholangitis via the VDR-miRNA155-SOCS1 Pathway. Int J Mol Sci 18: 289. 10.3390/ijms18020289 PMC5343825 28146070

