# Supplementary material for: ApaI polymorphism of vitamin D receptor affects health-related quality of life in patients with primary sclerosing cholangitis
Source: PLoS One. 2017 Apr 20;12(4):e0176264. doi: 10.1371/journal.pone.0176264 (PMC5398696; doi:10.1371/journal.pone.0176264)
Supplement: S1 Table — (DOC) [file pone.0176264.s001.doc]

**S1 Table. Clinical and laboratory date depending an analyzed polymorphisms.**

| ***ApaI* (rs7975232)** | | | | | | |
| --- | --- | --- | --- | --- | --- | --- |
| **Feature** | **AA**  ***[AA]***  **(n=67)** | **AC**  ***[aA]***  **(n=124)** | **CC**  ***[aa]***  **(n=84)** | ***P****  ***(AA vs aA)*** | ***P****  ***(aa vs aA)*** | ***P****  ***(aa vs AA)*** |
| **Gender (F/M)** | 22/45 | 41/83 | 30/54 | 0.9 | 0.8 | 0.7 |
| **Age of diagnosis (years)** | 30.2±1.5 | 31.2±1.2 | 31.2±1.5 | 0.6 | 1.0 | 0.6 |
| **Cirrhosis (No/Yes)** | 44/23 | 83/41 | 54/30 | 0.8 | 0.8 | 0.9 |
| **Haemoglobin (IU/ml)** | 13.1±0.3 | 13.2±0.5 | 13.4±0.2 | 0.8 | 0.7 | 0.6 |
| **AST (IU/ml)** | 131±20 | 136.1±16.7 | 141.2±25.1 | 0.8 | 0.9 | 0.7 |
| **ALT (IU/ml)** | 182.2±26.8 | 200.2±19.7 | 179.9±21.3 | 0.6 | 0.5 | 0.9 |
| **AP (IU/ml)** | 424.2±48.4 | 475.6±34.8 | 381.9±27.5 | 0.3 | 0.06 | 0.5 |
| **GGT (IU/ml)** | 381.9±41.2 | 472.9±44.3 | 381.3±33.9 | 0.1 | 0.1 | 1.0 |
| **Bilirubin (mg/dl)** | 3.2±0.7 | 2.9±0.4 | 3.1±0.6 | 0.7 | 0.8 | 0.9 |
| **Cholesterol (mg/dl)** | 217.9±11.8 | 235.6±13.8 | 198.7±8.2 | 0.3 | 0.05 | 0.3 |
| **TG (mg/dl)** | 100±11.1 | 102.5±7.8 | 100.8±12.7 | 0.9 | 0.9 | 1.0 |
| ***BsmI* (rs15444410)** | | | | | | |
| **Feature** | **AA**  ***[BB]***  **(n=40)** | **GA**  ***[bB]***  **(n=121)** | **GG**  ***[bb]***  **(n=114)** | ***P****  ***(BB vs***  ***bB)*** | ***P****  ***(bb vs bB)*** | ***P****  ***(bb vs BB)*** |
| **Gender (F/M)** | 8/32 | 47/74 | 38/76 | 0.05 | 0.4 | 0.1 |
| **Age of diagnosis (years)** | 29.8±2.0 | 30.4±1.1 | 31.9±1.3 | 0.4 | 0.8 | 0.4 |
| **Cirrhosis (No/Yes)** | 27/13 | 83/38 | 71/43 | 0.9 | 0.4 | 0.7 |
| **Haemoglobin (IU/ml)** | 13.5±0.3 | 12.8±0.2 | 13.7±0.6 | 0.09 | 0.8 | 0.3 |
| **AST (IU/ml)** | 134.6±30.5 | 135.8±14.6 | 138.5±18.9 | 0.9 | 0.9 | 1.0 |
| **ALT (IU/ml)** | 187.2±38 | 181.9±17.6 | 198.7±20.5 | 0.5 | 0.8 | 0.9 |
| **AP (IU/ml)** | 422.5±54.9 | 477.8±37.4 | 392.3±25.7 | 0.06 | 0.6 | 0.4 |
| **GGT (IU/ml)** | 434.8±78.1 | 455.5±41.3 | 385±30.5 | 0.2 | 0.5 | 0.8 |
| **Bilirubin (mg/dl)** | 2.8±0.8 | 2.9±0.4 | 3.2±0.5 | 0.6 | 0.6 | 0.9 |
| **Cholesterol (mg/dl)** | 213.1±14.1 | 228.4±10.3 | 213.4±12.9 | 0.3 | 1.0 | 0.5 |
| **TG (mg/dl)** | 100.3±10.7 | 95.3±7.6 | 108.3±10.7 | 0.3 | 0.6 | 0.8 |
| ***TaqI* (rs731236)** | | | | | | |
| **Feature** | **TT**  ***[TT]***  **(n=116)** | **TC**  ***[Tt]***  **(n=124)** | **CC**  ***[tt]***  **(n=35)** | ***P****  ***(TT vs***  ***Tt)*** | ***P****  ***(tt vs***  ***Tt)*** | ***P****  ***(tt vs***  ***TT)*** |
| **Gender (F/M)** | 40/76 | 45/79 | 8/27 | 0.8 | 0.1 | 0.2 |
| **Age of diagnosis (years)** | 31.8±1.3 | 30.8±1.1 | 28.5±2.2 | 0.5 | 0.3 | 0.2 |
| **Cirrhosis (No/Yes)** | 71/45 | 87/37 | 22/13 | 0.2 | 0.4 | 0.9 |
| **Haemoglobin (IU/ml)** | 13.7±05 | 12.8±0.2 | 13.5±0.3 | 0.9 | 0.4 | 0.8 |
| **AST (IU/ml)** | 135.9±19 | 144.1±16.9 | 112.4±16 | 0.7 | 0.4 | 0.5 |
| **ALT (IU/ml)** | 188.6±19 | 201.5±21 | 152.6±23 | 0.6 | 0.2 | 0.4 |
| **AP (IU/ml)** | 406.3±26.9 | 471.8±37.2 | 400.3±54.3 | 0.1 | 0.3 | 0.9 |
| **GGT (IU/ml)** | 397.3±30.6 | 467.1±44.4 | 355±55 | 0.2 | 0.1 | 0.6 |
| **Bilirubin (mg/dl)** | 3.3±0.5 | 2.9±0.4 | 2.5±0.9 | 0.6 | 0.7 | 0.4 |
| **Cholesterol (mg/dl)** | 213.1±12.9 | 231.1±10 | 202±12.8 | 0.2 | 0.2 | 0.6 |
| **TG (mg/dl)** | 108.5±10 | 96.3±7.6 | 95.9±13.8 | 0.3 | 1.0 | 0.5 |

* ANOVA with Fisher's protected least significant difference (PLSD);

AST: Aspartate aminotransferase; ALT: Alanine aminotransferase;

ALP: Alkaline Phosphatase; GGT: Gamma-glutamyl transferase
